# Monte Carlo modelling of experimental setup used for biodosimetry intercomparison

**DOI:** 10.1093/rpd/ncad166

**Published:** 2023-09-18

**Authors:** Dinindu Gunasekara, Ruth Wilkins, Frédéric Tessier, Lindsay Beaton-Green

**Affiliations:** Environmental and Radiation Health Sciences Directorate, Health Canada 775 Brookfield Rd, Ottawa K1A 1C1, Canada; Environmental and Radiation Health Sciences Directorate, Health Canada 775 Brookfield Rd, Ottawa K1A 1C1, Canada; Ionizing Radiation Standards, National Research Council Canada 1200 Montréal Rd, Ottawa K1A 0R6, Canada; Environmental and Radiation Health Sciences Directorate, Health Canada 775 Brookfield Rd, Ottawa K1A 1C1, Canada

## Abstract

When using biodosimetry techniques to assess absorbed dose from an ionising radiation exposure, a calibration curve is required. At Health Canada (HC), these curves are generated for a variety of radiation qualities and assays to translate biological damage into absorbed dose. They are produced by irradiating biological samples in custom-designed water-equivalent phantoms inside a cabinet X-ray machine. In the HC lab, two different phantoms can be used for irradiation that differs in material composition and internal geometry. To ensure consistency, the impact of using the phantoms interchangeably was investigated. This was done through lab measurements and the development of a Monte Carlo (MC) model. Differences up to 6.7% were found between the two experimental setups, indicating the need for careful consideration if using these setups interchangeably in the laboratory. Once validated, the MC model can be used to investigate different aspects of the experimental setup without the need for laboratory measurements.

## Introduction

Exposure to ionising radiation can lead to adverse health effects in human beings. Therefore, accurately measuring the level of ionising radiation to which one is exposed is important. The amount of radiation received by specific individuals can be assessed through the use of biodosimetry (BD)^(^^1^^)^. This refers to techniques that quantify the level of biological damage in an individual and converts this to an amount of dose absorbed by that individual. For example, by correlating the number of chromosome aberrations induced after irradiation to the dose used to produce that damage, one can, in principle, determine an unknown dose to an individual. This correlation comes in the form of a dose–response calibration curve. At Health Canada (HC), these curves are produced by irradiating biological samples in custom-designed water-equivalent phantoms inside a cabinet X-ray machine. When collecting the data to build these curves, it is important to carefully consider the experimental setup as this can affect the accuracy of the curve. In the HC lab, two different phantoms are used that vary in terms of their internal geometries and their material composition. Analyzing the difference in ion chamber output when placed in these phantoms can indicate whether the phantoms can be used interchangeably, or if there is a significant influence on the dose that needs to be considered.

While the differences between these phantoms can be explored through measurements in the laboratory, an alternative strategy is to use computational simulations. Monte Carlo (MC) computational methods can predict dose by computing energy deposition from a very large ensemble of radiation tracks modelled in detail, based on known radiation transport cross sections. Since the exposure system involves an X-ray source, the EGSnrc MC toolkit^(^^2^^)^ was chosen to create the model because of its well-established accuracy and efficiency in photon and electron transport simulations. In addition, it includes several variance reduction techniques that can further boost simulation efficiency. The model can be validated using laboratory measurements, and its applications can then be extended beyond the comparison of these two phantoms. Once validated, computational simulations become versatile tools as one can change different aspects of the simulation environment to explore factors that could influence laboratory results, without the need for experimental measurements. With this in mind, the validated MC model can be used to further investigate aspects of the experimental setup such as the positioning of the ion chamber in the field, the effect of irradiating multiple samples simultaneously and other factors that could affect the dose output.

This paper describes the MC code that was developed to model the HC X-ray system. It further describes how the MC model was then used to compare two different experimental setups used at HC for biological irradiation through laboratory measurements.

## Materials and methods

The setup at HC used to irradiate blood samples consists of three major components that must be modelled: the cabinet X-ray machine, the ionisation chamber and the two water-equivalent phantoms. The source of radiation used to irradiate blood samples is an X-ray photon source, produced by the XRAD-320 cabinet X-ray machine (XRAD, Precision X-ray, Madison, United States). The XRAD is capable of delivering a maximum X-ray energy of 320.0 keV and a maximum X-ray tube current of 15.00 mA. In addition to the 2 mm of inherent beryllium filtration attenuating the beam, measurements were made with three different beam filter configurations:

- Filter 1: 2.0 mm aluminum
- Filter 2: 1.5 mm aluminum +0.25 mm copper +0.75 mm tin
- Filter 3: 2.5 mm aluminum +0.13 mm copper

**Figure 1:**
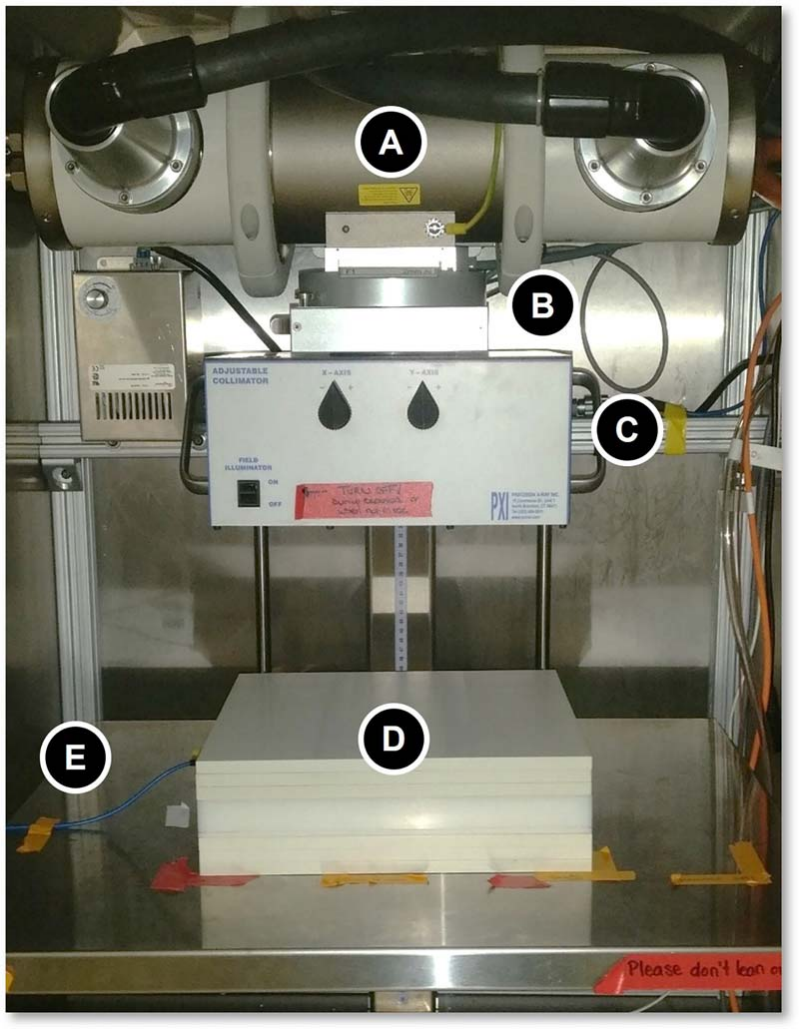
A view of the inside of the Precision XRAD-320 cabinet X-ray machine with the ion chamber in place within the solid water phantom. The setup consists of the following components: **A**. X-ray tube, **B**. Primary collimator, adjustable filter and monitor chamber, **C**. Mirror, collimating jaws, **D**. Solid water phantom, **E**. Ion chamber cable connecting to ion chamber inside the phantom.

Accumulated charge measurements (in nC) were taken using the PTW TW30010 (PTW, Freiburg, Germany) ionisation chamber in conjunction with the PTW UNIDOS T10002 (PTW, Freiburg, Germany) electrometer as an analog to absorbed dose. For all measurements in this study, exposure time was 2 minutes, which corresponded to charge accumulation rates from 3.32 to 35.66 nC/min depending on phantom used, amount of filtration and beam energy. The ionisation chamber was calibrated by the Measurement Science and Standards group at the National Research Council Canada (NRC), 8 May 2020. The calibration coefficient provided by the NRC was 48.07 mGy/nC for a generating potential of 250 kV, meaning the dose rates were in the range of 0.16–1.71 Gy/min. For each measurement, the ion chamber was placed inside the solid water phantoms that are square slabs of side length 15 cm and various thicknesses (0.1–3.0 cm). An image of the experimental setup can be seen in Figure 1, where the main components of the XRAD that were modelled are labelled, along with the ion chamber and phantom in beam path. In addition, uncertainties on all lab measurements were estimated by considering sources of error from both the instruments and the experimental setup. This was accomplished by combining the error from setting the source-to-surface distance (SSD) with the error from setting the field size. The statistical error from the lab measurements was also consider, but it was found to be too small in relation to the setup errors. Both the SSD and field size errors were estimated by measuring dose values at a variety of different SSDs and field sizes around the values of interest to the actual measurements. By quantifying the dose variation across these distances and field sizes, a dose output error value could be associated with the uncertainty in setting the SSD and field size.

**Figure 2:**
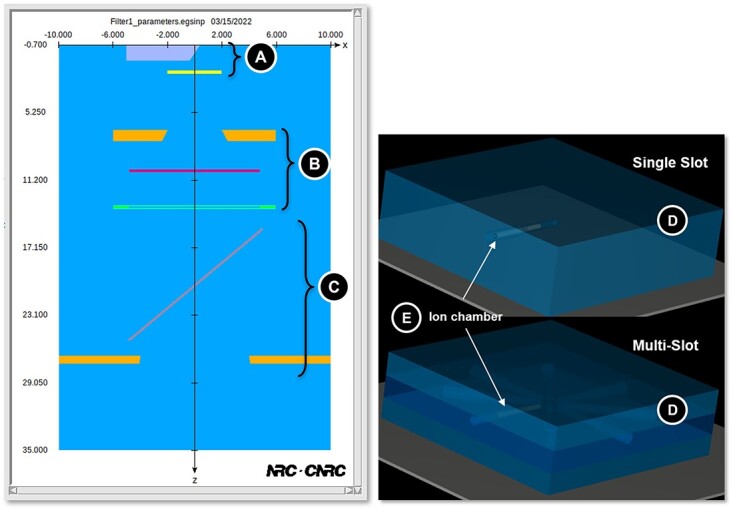
Schematic of the Monte Carlo model of the XRAD created in the EGSnrc application, BEAMnrc. All components labelled in Figure 1 are labelled here, with the phantom and the ion chamber are modelled separately in egs_chamber. In addition, both the single slot (top right) and multi-slot (bottom right) are shown with the location of the ion chamber labelled in both phantom models.

To replicate this setup in the form of an MC simulation, the EGSnrc toolkit was used. More specifically, the applications *egs_chamber*^(^^3^^)^ and *BEAMnrc*^(^^4^^)^ were used to create two different models of the setup. The first of these models, called the ‘BEAMnrc Model’, uses the application BEAMnrc to simulate the XRAD-320 itself and uses this as a source in the application egs_chamber where the ion chamber and phantom geometries were modelled. Specifications were derived through assistance from the manufacturer, as well as careful examination of previous work done by Azimi et al.^(^^5^^)^ and Lee & Ye^(^^6^^)^. The view of the model of the XRAD-320 from the BEAMnrc graphical user interface is shown in the left of Figure 2, and the individual components (A, B, C) of the X-ray setup shown in Figure 1 are equivalently labelled in the MC model. Notably, the ion chambers have been explicitly labelled in Figure 2 as they can be seen more clearly than in Figure 1. To improve the efficiency of the simulations in the XRAD, two variance reduction techniques were optimised and used in the BEAMnrc code: directional bremsstrahlung splitting (DBS) was enabled alongside bremsstrahlung cross-section enhancement (BCSE) to offset the inefficiency of bremsstrahlung production in the X-ray target at these energies. For these techniques, the parameters were optimised with the BCSE factor set to 100 and a splitting field radius of 12 cm. The optimal splitting number was found to be approximately 100,000. The BEAMnrc input file of the XRAD was then used as the source of X-ray photons in the egs_chamber application. The second model, called the ‘SpekPy Model’, replaces the entire X-ray tube portion of the XRAD-320 with an equivalent spectral point source of photons. The source spectrum is created using *SpekPy*^(^^7^^)^, markedly increasing the efficiency of the simulation, bypassing the explicit modelling of the inefficient bremsstrahlung conversion process. The rest of the XRAD-320 is modelled in egs_chamber using egs++, and a set of C++ class libraries implemented in EGSnrc to define elaborate simulation geometries. Typically, a simulation consisted of at least 10^9^ source particles, with all values being compared in a ratio always having the same number of source particles in their respective simulations.

The phantoms and the ionisation chamber were also modelled using egs++ geometry in egs_chamber. Two different types of solid water phantoms were modelled, one with a single slot for either a blood vial or the ion chamber and the other with eight slots, shown on the right side of Figure 2, allowing for the irradiation of seven blood vials and the ion chamber simultaneously. Notably, the multi-slot phantom contains a layer of a water-equivalent material polystyrene in the middle, instead of being fully composed of solid water like the single-slot phantom. Two additional variance reduction techniques were used, namely photon cross-section enhancement (XCSE), and range-based Russian roulette (RR), to improve the efficiency of the simulation. An enhancement factor of 512 was used in photon XCSE, along with a rejection factor of 512 for RR. A thin cylindrical shell was defined around the air gap containing the ion chamber, and every region therein was enhanced with XCSE.

To compare the two setups used for irradiating blood samples, the ratio of the dose output from the ion chamber using the two phantoms was measured in the lab and calculated in EGSnrc for each model. To examine the difference between the setups, multiple filter configurations were used in the X-ray cabinet, and ratios were calculated for each filter defined previously. An additional form of validation that is standard for X-ray beams is the determination of the half-value layer (HVL) of a given material. The HVL refers to the thickness of a material that reduces the incoming radiation intensity by half. For this study, different thicknesses of copper (0.5, 1.0, 2.0 and 2.5 mm) were placed in the beam path by securing the piece of metal to the bottom of the XRAD-320 exit window. Measurements were taken with filter 1 in place. In addition, a measurement was taken with no copper in place to which the rest of the measurements could be normalised. These measurements were then plotted and fit using MATLAB R2021b, and the thickness at which the electrometer reading dropped to a relative value of 0.5 was calculated. Due to this not being a monoenergetic beam, the attenuation curve could not be modelled by a simple exponential, and the curves were fit with a more complicated function to find the HVL. The exact same setup was recreated in the EGSnrc models but was carried out with a larger number of thicknesses (0.05–2.5 mm).

## Results and discussion

**Figure 3:**
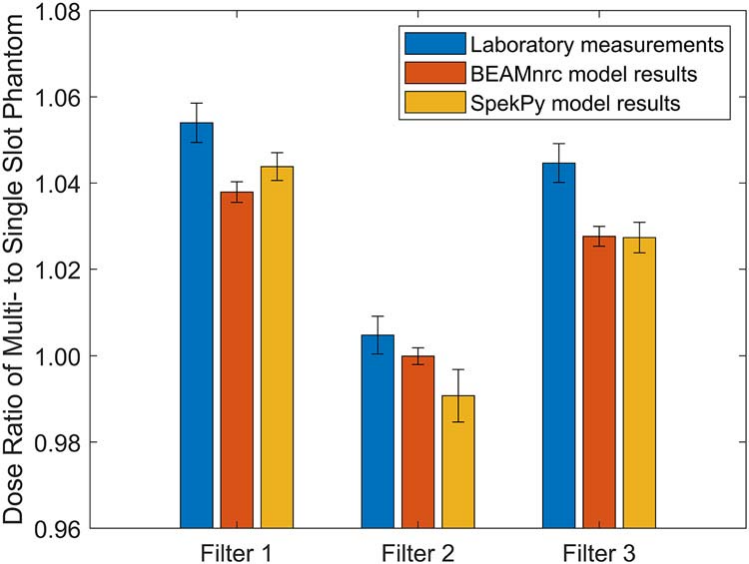
Comparing different phantom setups using dose ratios: graphical summary of the ratio of the dose output of the ion chamber in the multi-slot phantom to the single-slot phantom. Error bars are displayed for both measured and simulated ratio values representing a standard deviation above and below each ratio. The measured error values take into account experimental setup error, while the simulated error values are purely statistical errors based on the number of particles simulated.

Radiation dose output ratios between the ion chamber in the multi-slot and the single-slot phantoms were measured in the lab and simulated in EGSnrc for all three filters and represented in Figure 3. The lab results were compared to the output of each of the simulations by taking the difference between them and dividing by their combined uncertainty. This provided a measure of the number of standard deviations each simulation result differed from their respective lab measurement, with a difference of less than 2 standard deviations being considered acceptable agreement. For the BEAMnrc model, the ratio for filter 2 was found to be in agreement with lab measurement, while the other two filters were not. The SpekPy model provided ratios for filters 1 and 2 that were in agreement lab measurements, while the filter 3 ratio was not. The general trend between both models was that they consistently underestimated the dose output when compared to the equivalent lab measurements, with the SpekPy model yielding better results. The most likely cause of discrepancy between the simulations and laboratory measurements is an inaccuracy in the assumptions made for the specifications of the model. Mainly this includes the lack of knowledge on the spacing of the different components along the beam path, as well as not accounting for the backscatter from the enclosure between the monitor chamber and the jaws. These geometrical issues may result in the energy spectrum of the X-ray beam in the simulation differing from the spectrum of the lab beam due to the difference in attenuation along the beam path. This could cause an underestimation of the output by the simulation depending on how the spectra differ. Although this cannot be truly confirmed without measuring the output spectrum in the lab, it appears to be the most likely explanation for the discrepancy. Another possibility is due to the age of the XRAD, the filter might have deteriorated enough to affect the dose being output, causing a mismatch between the simulation and lab. Comparing between simulations, the expectation was that BEAMnrc would agree better with lab measurements due to the much more accurate geometrical modelling of the X-ray production, but this is not the result found in this study. Due to the SpekPy model originating from a point source, there would be fewer photons scattered away from passing through the sensitive region of the ion chamber than for the BEAMnrc model. This would cause an increase in the dose recorded from the SpekPy model and might explain the discrepancy seen between the models.

Using the ratios to compare the two phantoms, the largest difference was for filter 1, where the multi-slot phantom increased the dose by around 6.7%. In contrast, the smallest difference was for filter 2 with around 0.48% higher dose being found in the multi-slot phantom. Although almost all of the results from both lab and simulation agreed that using the multi-slot phantom increases the overall dose deposited. Initially, this seems counterintuitive because the multi-slot phantom contains more gaps of air, and hence less solid water surrounding the ion chamber for photons to scatter into the sensitive region and increase the measured dose. However, this can be explained by considering the composition of the phantoms in question. The single slot phantom consists entirely of RW3, while the multi-slot phantom has a 3 cm layer of polystyrene in the middle of the phantom with the rest being RW3. Both materials are water equivalent, but they are slightly different in that polystyrene is approximately 1.5% more dense than RW3^(^^8^^)^. When considering ratios very close to unity, this type of disparity can cause the ratios to flip.

**Figure 4:**
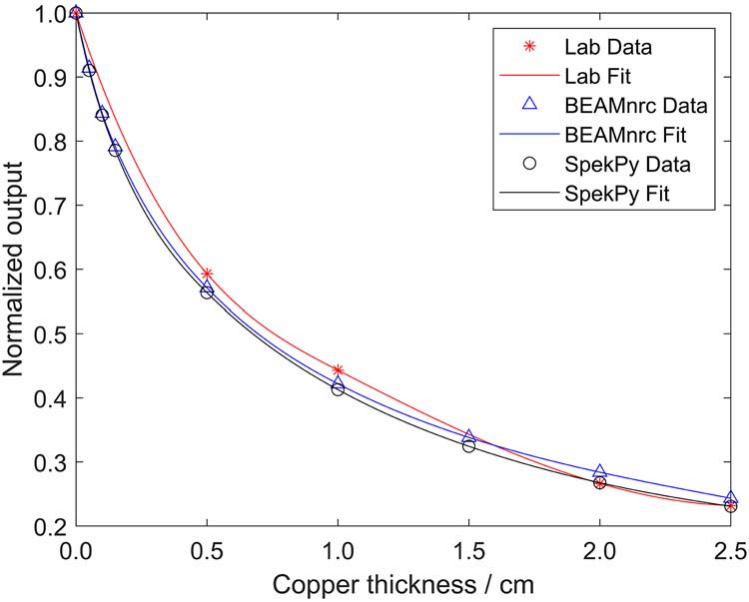
Attenuation curve plotted for copper to compare laboratory measurements to MC model output. Each set of points was fit using a cubic spline in MATLAB R2021b and the half-value layer was calculated for each curve. Error bars are too small to be visualised. The error on the lab data is based on experimental setup error, while the error on the simulated data is statistical error.

As an additional method of validation, the HVL of copper was determined. The attenuation data from the laboratory and each of the EGSnrc models are plotted in Figure 4, and each data set was fit with a cubic spline function in MATLAB R2021b. The HVL calculated was 0.5931 mm for the laboratory measurements, 0.5711 mm for the BEAMnrc model data and 0.5635 mm for the SpekPy model data. The BEAMnrc HVL differed from the lab value by 3.71%, while the SpekPy HVL differed by 4.99%. Looking at the curves, it appears to that, in general, lab measurements predict a higher output until the thicknesses of copper exceeds 1.5 mm and then the simulations predict a higher dose. The trends with thin copper are in line with previous observations that the models underestimate the dose. To account for the trends with thicker copper, similar arguments as earlier can be made about the inaccuracies in the model with regard to backscatter and component positioning. A combination of errors can result in either an increase or a decrease in the relative dose output measured. Overall, the discrepancy in HVL calculations between lab and simulations is relatively larger than the discrepancies seen for the phantom output ratios. This further indicates that the issue lies within in the internal geometry definitions of the XRAD-320.

With the MC model now established with a good degree of accuracy for low levels of filtration, it can be used to compare variations of other aspects of the experimental setup at HC. One example is varying the position of the ionisation chamber in terms of its distance from the center of the field. This type of analysis could be done with relative ease in EGSnrc as it involves changing only a few parameters that define the position of the geometry that creates the ion chamber and rerunning the simulation. In general, any change can be made to the overall simulation geometry to explore any setup variation of interest much faster than one could analyze the same scenario with lab measurements.

## Conclusions

From both the lab measurements and simulations, it was found that the choice of phantom configuration does influence dose, but in all cases this effect is relatively small, especially when considering a highly attenuating filter. Using the multi-slot phantom increases the amount of dose absorbed when compared to the single-slot phantom. There remain some issues with both models consistently underestimating the dose output that could likely be addressed through more accurate modelling around the X-ray beam path. Additional validation of the models through the calculation of the HVL of copper showed discrepancies relatively larger than those seen for the phantom ratios, further justifying the need for investigating of the materials in the beam path. The next step in this project is to finalise the validation of this X-ray setup model. Once complete, this model can be used in conjunction with TOPAS-nBio, an MC software that specialises in modelling biological damage, to calculate dose–response curves.

## Data Availability

The data underlying this article will be shared on reasonable request to the corresponding author.
